# The effects of supplemental brewers yeast on postprandial amino acid concentrations in healthy adult sled dogs

**DOI:** 10.1093/jas/skaf180

**Published:** 2025-05-24

**Authors:** Lindsey M Rummell, James R Templeman, Cara L Cargo-Froom, Anna K Shoveller

**Affiliations:** Department of Animal Biosciences, Ontario Agricultural College, University of Guelph, Guelph, Ontario, Canada N1G 2W1; Wilbur Ellis Nutrition, Buhl, ID 83316, USA; Department of Animal Biosciences, Ontario Agricultural College, University of Guelph, Guelph, Ontario, Canada N1G 2W1; Department of Animal Biosciences, Ontario Agricultural College, University of Guelph, Guelph, Ontario, Canada N1G 2W1; Department of Animal Biosciences, Ontario Agricultural College, University of Guelph, Guelph, Ontario, Canada N1G 2W1

**Keywords:** beta-glucan, canine, inflammatory status, meal challenge, postprandial amino acid appearance, *Saccharomyces cerevisiae*

## Abstract

Yeast has been used in animal systems to modulate the immune response and support gut health. Certain amino acids (**AA**) are reported to also exert positive effects on the gut, supporting the intestinal barrier and restoring mucosal immune homeostasis. The objective of this study was to evaluate the effects of brewers yeast on postprandial serum AA appearance in dogs. Nineteen privately owned domestic Siberian huskies and 1 Alaskan husky (9 females: 5 intact, 4 spayed; 11 males: 3 intact, 8 neutered), with an average age of 4.8 ± 2.6 yr and body weight (**BW**) of 25.6 ± 4.1 kg, were enrolled in this study. Ten dogs received a dry extruded control diet (Ctl) and 10 the Ctl diet top-dressed with yeast to receive a daily ß-glucan dose of 7 mg/kg BW (treatment, Trt) for 10 wk. At weeks −1, 2, 4, and 8 postprandial free AA concentrations were quantified. A fasted blood sample was collected, a meal was provided, followed by further blood sampling 1, 2, and 4 h post-meal. Serum IL-10 concentrations were analyzed from the fasted sample. All data were analyzed using PROC GLIMMIX of SAS, with dog as a random effect and week, diet group, and sampling time point as fixed effects for AA, and with dog as a random effect and week as a fixed effect and repeated measure for IL-10. There was no effect of treatment on any AA, though a significant Trt*wk*timepoint interaction effect was observed for His and Trp (*P *≤ 0.05). An overall increase was observed in many AAs for all dogs—serum concentrations of Lys, Met, Thr, Trp, Ala, Arg, Gln, Gly, Pro, Ser, Tau, and Tyr were greatest at week 8 in all dogs when compared to concentrations at week −1 (*P *≤ 0.05). Serum IL-10 concentrations did not differ by week or between groups (*P* > 0.05). In 3 Trt dogs that had greater gut permeability than all other dogs at week −2, as reported in a previous publication, serum Ile, Thr, and Val concentrations were greater at week 8 compared to week −1 (*P *≤ 0.05) and Leu concentrations were greater 1 and 2 h postprandial at week 8 compared to the same time points at week −1 (*P *≤ 0.05). The results of this study suggest that supplemental yeast may have a beneficial effect on peripheral AA availability without impacting inflammatory status in dogs. Future studies should seek to investigate the effects of reducing or improving gut permeability with yeast on nutrient digestive and metabolic efficiencies or consider yeast in clinical nutrition to support dogs with gastrointestinal diseases.

## Teaser

This study investigated the effects of a yeast supplement on the post-meal appearance of amino acids in the serum of dogs. Overall, yeast supplement had no detrimental effects on healthy dogs and increased post-meal serum amino acid appearance in dogs with elevated gut permeability, supporting its inclusion in canine diets.

## Introduction

Yeast is an ingredient commonly utilized in pet food. The effects of yeast in many animal models have been extensively evaluated, though results differ depending on yeast source, preparation, and dose. Yeast has protective effects in the gastrointestinal tract (**GIT**), including supporting maintenance of intestinal barrier integrity in mice, as well as stimulating the immune system and preventing translocation of bacteria and endotoxins from the gut into portal and systemic circulation (Generoso et al., 2011). These immune modulating and gut stimulating effects make yeast an appealing ingredient for use in companion animal diets. While yeast is made up of a variety of biologically active compounds, such as B-vitamins, antioxidants, mannooligosaccharides, fructooligosaccharides, and ß-glucans, the ß-glucan fraction has been reported to act as an immune modulator and to exert protective effects in the canine gut (Martins et al., 2014).

A cell wall component of yeast, fungi, cereal grains, algae, lichen, and some bacteria, ß-glucans vary in structure and function based on source (Novak and Vetvicka, 2009). Yeast-derived ß-glucans act as immunostimulants in mice (Lee et al., 2001) and dogs (Stuyven et al., 2010). ß-glucans trigger the release of nitric oxide, arachidonic acid metabolites, and cytokines (Jung et al., 2004; Suram et al., 2006; Chen and Seviour, 2007) by binding to specific receptors on immune cells such as macrophages, neutrophils, monocytes, dendritic cells, and natural killer cells (Stuyven et al., 2010). While yeast may offer benefits to gut health, other ingredients and many nutrients also convey beneficial effects when fed alone on in combination with yeast.

Dietary amino acids (**AA**) are essential for the maintenance of health in dogs. A number of AA have ameliorative effects on intestinal growth and support the maintenance of the intestinal barrier function in dogs and other species (Liu et al., 2017; Benvenuti et al., 2020). Among these, Thr and Gln are most notable for their ameliorative effects on gut barrier integrity and function, Thr through incorporation into mucins (Floc’h and Sève, 2005; Schaart et al., 2005), and Gln as a precursor for the synthesis of antioxidants and cytokines (Field et al., 2002). Additionally, Arg, Gln, Gly, Cys, and Pro function in the GIT of dogs and other species to mitigate damage to the gut, support the integrity and function of the intestinal barrier, reduce oxidative stress, maintain mucosal immune homeostasis, and enhance the GIT by attenuating the secretion of inflammatory cytokines, such as interleukin (**IL**)-6, and increasing concentrations of immune-regulating cytokines such as IL-10 (Ruth and Field, 2013; Li et al., 2016; Liu et al., 2017; He et al., 2018; Kathrani et al., 2018). Studies in pigs reported increased apparent ileal digestibility (**AID**; Wu et al., 2018; Park et al., 2021), and increased concentrations of various AAs in serum (Xiong et al., 2015) of pigs receiving supplemental yeast compared to control. The improved digestibility reported in these pigs is likely the cause of the greater postprandial AA appearance, as digestibility of nutrients is influenced by both gut function and the ingredient itself (Xiong et al., 2015). While measuring AID provides a more accurate assessment of peripheral AA availability, this method is not utilized in dogs due to ethical constraints. As such, we have chosen to approach this problem by quantifying the appearance of AA in the peripheral circulation as a proxy for measuring the AID of AA. While there is considerable research into the roles of AA in human models of intestinal inflammation, little research has investigated these metabolic mechanisms in dogs.

In order for nutrients to be utilized, they must be digested and subsequently absorbed into circulation (Stein et al., 2007). When intestinal barrier function is impaired, this can negatively affect uptake of AA and other nutrients as the intestinal barrier plays a critical role in this process. In contrast, when gut health is maintained or restored, this enhances the surface area for nutrient absorption and can increase circulating AA (Habashy et al., 2017). The immune modulating and gut stimulating effects of yeast and certain bioactive components have been well-documented in pigs (Che et al., 2017)  and has been studied in dogs (Stuyven et al., 2010, 2010; Beloshapka et al., 2013; Rychlik et al., 2013), though results are dependent upon strain, preparation, and dose. The overall suggestion that yeast can improve gut health, and potentially nutrient absorption by extension, warrants additional investigation.

Though the immunomodulatory effects of yeast have been well-documented in both animal and human models (Lee et al., 2001; Stuyven et al., 2010; Vich Vila Arnau et al., 2018), there is limited research on how yeast supplementation affects AA concentrations. Additionally, though the roles of AA in human models of intestinal inflammation have been well-documented (Li et al., 2007; Landy et al., 2016; Bao et al., 2017; He et al., 2018; Vich Vila Arnau et al., 2018), there are limited data for these mechanisms in dogs. As such, the objective of this study was to evaluate the effects of a concentrated brewers yeast product on serum postprandial AA concentrations and fasted serum IL-10 concentrations in healthy adult dogs. We hypothesized that dogs supplemented with brewers yeast would have greater postprandial serum AA concentrations, and greater concentrations of the anti-inflammatory cytokine IL-10 compared to control dogs.

## Materials and Methods

### Animals and housing

This study was approved by the University of Guelph’s Animal Care Committee (Animal Use Protocol # 4412) and was in accordance with national and institutional guidelines for the care and use of animals in research. Nineteen privately owned domestic Siberian huskies and 1 Alaskan husky (9 females: 5 intact, 4 spayed; 11 males: 3 intact, 8 neutered), with an average age of 4.8 ± 2.6 yr and body weight (**BW**) of 25.6 ± 4.1 kg (mean ± SD), were used in this study. The study took place between August and November 2020. Dogs were housed at an off-site facility (Rajenn Siberian Huskies, Ayr, ON) that was previously visited and approved by the University of Guelph’s Animal Care Services. Throughout the study, dogs were housed in free-range, outdoor kennels ranging in size from 3.5 to 80 square meters. Each kennel contained between 2 and 9 dogs. Dogs had ad libitum access to fresh water, constant access to shelter, received daily socialization, and the same amount of weekly exercise. Dogs were weighed weekly, and feed intake was adjusted to maintain week 0 (baseline) BW. All dogs remained healthy throughout the study period. Complete blood count and serum biochemistry values are reported in Rummell et al. (2022).

### Diet and study design

For complete diet and study design, refer to Rummell et al. (2022). Briefly, dogs were blocked by age, sex, and BW prior to being randomly allocated to 1 of 2 diet groups: control (Ctl; *n* = 10; 5 males, 4 neutered, 1 intact; 5 females, 3 spayed, 2 intact; 4.80 ± 2.82 yr average age, 25.43 ± 3.65 kg average BW) or treatment (Trt; *n* = 10; 6 males, 4 neutered, 2 intact; 4 females, 1 spayed, 3 intact; 4.80 ± 2.62 yr average age, 25.40 ± 4.47 kg average BW).

All dogs were acclimated to a Ctl diet (Acana Adult Large Breed, Champion Petfoods LT, Morinville, AB) from week −4 to −1 (4 wk). The Ctl diet met or exceeded all National Research Council (National Research Council, 2006) and Association of American Feed Control Officials (Association of American Feed Control Officials, 2016) nutrient recommendations for adult dogs. For additional details regarding the nutrient composition of the Ctl diet, refer to Rummell et al. (2022).

Following the diet acclimation period, Ctl dogs continued to receive the Ctl diet from weeks 0 to 9, while dogs on Trt were fed the Ctl diet top-dressed with brewers dried yeast (Wilbur Ellis Nutrition, Buhl, ID, USA). The total period of supplementation was 10 weeks. Yeast was supplemented to provide a ß-glucan dose of 7 mg/kg BW per day based on a previous study in dogs (Rychlik et al., 2013), resulting in Trt dogs receiving 0.2 g of yeast per kg BW daily. All dogs were fed once daily individually to allow for the monitoring of food consumption and proper allocation of dietary treatments. Any orts (offered, refused, treatment) were weighed and recorded daily. Feed intake was initially determined based on historical feeding records and adjusted weekly to ensure all dogs maintained their initial BW throughout the study. Data for mean daily feed intake and BW are reported in Rummell et al. (2022).

### Blood sample collection and analysis

Postprandial serum free AA concentrations were evaluated on weeks −1, 2, 4, and 8. Dogs were fasted overnight for 12 h and a 5 mL fasted blood sample was collected via cephalic venipuncture with a serum Vacutainer (Becton, Dickinson and Company, Franklin Lakes, NJ, USA) using a winged infusion set (Terumo Surflo Winged Infusion Set, 21 G x.75, Terumo Medical Corporation, Vaughn, ON). Immediately following the fasted sample, dogs received a meal consisting of 75% of their daily ration followed by postprandial blood collections at 1, 2, and 4 h postprandial, as described above. All samples were centrifuged at 2,000 x *g* for 20 min at 4 °C using a Beckman J6-MI centrifuge (Beckman Coulter, Indianapolis, IN), then serum aliquots were collected, frozen, and kept at −80 °C prior to analysis. Serum samples were analyzed for free AA using an ultra-performance liquid chromatography system (UPLC; Waters Corporation, Milford, MA) using the method described by Templeman et al. (2020). Total serum Cys, Hcys, and free GSH were analyzed with UPLC using the methods described by Banton et al. (2021). Serum IL-10 concentrations were evaluated from the fasted sample using a solid-phase Canine Quantikine enzyme-linked immunosorbent assay (ELISA) (R&D Systems, Minneapolis, Minnesota, USA). Three other markers of inflammation and oxidative status, serum amyloid A, serum haptoglobin, and serum malondialdehyde, were analyzed and included as part of a previously published portion of this study (Rummell et al. 2022).

### Case study

Data previously published from this study suggested that brewers yeast-supplemented to dogs restored intestinal permeability, as evaluated using the intestinal permeability markers iohexol and chromium-ethylenediamine tetra-acetic acid (Rummell et al., 2022). Within the Trt group, 3 dogs (*n* = 3, female, average BW 22.3 ± 3.2 kg) with no physical indications (i.e., weight loss, loose stools) or known allergies had higher gut permeability when measured at week −2 in a previously published portion of this study that was ameliorated when re-measured at week 9 (Rummell et al., 2022). No other dogs in either group presented with elevated gut permeability. Postprandial AA appearance for these 3 dogs was compared at weeks −1 and 8. For the present data, we wanted to explore whether brewers yeast supplementation specifically improved the AA appearance in these 3 dogs to begin to explore the application of brewers yeast in dogs with poor gut health.

### Statistical analysis

Data were analyzed using SAS (v. 9.4; SAS Institute Inc., Cary, NC). Amino acid data were analyzed using PROC GLIMMIX of SAS where dog was treated as a random effect while week, diet group, and sampling time point were treated as fixed effects. Serum IL-10 data were analyzed using PROC GLIMMIX of SAS where dog was treated as a random effect, diet group as a fixed effect, and week as a fixed effect and repeated measure. For the case study, postprandial serum AA data were analyzed using PROC GLIMMIX of SAS where dog was treated as a random effect while week and sampling time point were treated as fixed effects. For each of the aforementioned procedures, when the main effects were significant means were compared using a Tukey’s HSD test. Significance was declared when *P* ≤ 0.05.

## Results

### Indispensable amino acids postprandial response

Mean serum indispensable AA (IAA) concentrations of Trt and Ctl at each week for all timepoints are reported in **Table 1**. There was no treatment or treatment*week interaction effect for any IAA (*P* > 0.05); however, a significant treatment*week*timepoint interaction effect was observed for His and Trp (*P* ≤ 0.05). Neither His nor Trp concentrations differed from fasted at any timepoint in week −1 in either group (*P* > 0.05). At week 2, serum concentrations of His and Trp in the Ctl group were not different from fasted at any timepoint (*P *> 0.05); however, serum concentrations of His in the Trt group were greater 4 h postprandial, and serum Trp concentrations in the Trt group were greater 2 and 4 h postprandial when compared to fasted (*P* ≤ 0.05). At week 4, serum Trp concentrations in the Trt and Ctl group were greater 1, 2, and 4 h postprandial when compared to fasted (*P* ≤ 0.05). Serum His concentrations at week 4 in the Ctl group were greater than fasted 2 and 4 h postprandial, and in the Trt group were greater than fasted 1, 2, and 4 h postprandial (*P* ≤ 0.05). Finally, at week 8, serum Trp concentrations were greater than fasted 1, 2, and 4 h postprandial in the Trt group, and in the Ctl group were greater than fasted 4 h postprandial (*P* ≤ 0.05). Serum His concentrations at week 8 in the Trt group were greater than fasted 2 and 4 h postprandial, and in the Ctl group were greater than fasted 4 h postprandial (*P* ≤ 0.05). No treatment*week*timepoint interaction effect was observed for any other IAA (*P* > 0.05).

**Table 1.** Mean serum concentrations of indispensable AA (IAA; nmol/mL) in control and yeast-supplemented dogs over a 10-week period

Amino Acid	Grp	Week −1					Week 2					Week 4					Week 8					*P*-value
		Fasted	1 h	2 h	4 h	SEM	Fasted	1 h	2 h	4 h	SEM	Fasted	1 h	2 h	4 h	SEM	Fasted	1 h	2 h	4 h	SEM	Wk*Trt*Time
Histidine	Trt	117	101	124	135	6	108	121	130	137^1^	6	96	121^1^	135^1^	136^1^	6	101	122	129^1^	129^1^	6	0.01
	Ctl	119	121	132	129	6	119	128	124	117	7	101	119	139^1^	143^1^	6	109	120	130	143.2^1^	6	
Isoleucine	Trt	66	71	83	105	7	73	87	101	113	7	83	133	150	142	7	74	107	119	131	7	0.61
	Ctl	64	67	83	101	7	80	91	84	87	8	84	130	144	136	7	71	98	117	132	8	
Leucine	Trt	122	125	143	168	12	130	150	171	182	12	160	242	263	237	12	146	191	208	215	12	0.78
	Ctl	124	126	146	167	12	144	157	150	149	14	166	236	255	233	12	133	171	196	214	13	
Lysine	Trt	116	148	170	186	14	114	160	178	190	15	90	180	226	218	14	106	187	207	204	14	0.5
	Ctl	124	153	177	187	15	160	183	170	183	16	94	190	240	244	15	116	201	224	231	15	
Methionine	Trt	50	56	59	67	3	52	64	65	69	4	47	62	74	82	3	51	66	70	78	3	0.77
	Ctl	57	59	63	70	4	61	68	67	65	4	50	64	75	89	4	55	66	73	85	4	
Phenylalanine	Trt	84	83	85	83	4	74	83	88	86	4	82	100	104	89	4	75	91	91	84	4	0.52
	Ctl	82	81	82	82	4	79	82	82	77	4	84	103	105	94	4	77	85	92	90	4	
Threonine	Trt	150	166	178	192	11	156	185	189	201	11	133	185	212	226	11	156	202	213	233	11	0.81
	Ctl	158	174	184	193	11	169	189	183	185	13	136	180	214	230	11	153	190	217	250	12	
Tryptophan	Trt	89	96	98	98	8	75	94	100^1^	108^1^	8	59^2^	83^1^	95^1^	106^1^	8	77	103^1^	105^1^	110^1^	8	0.02
	Ctl	89	94	91	96	8	87	97	80	82	8	57^2^	89^1^	96^1^	104^1^	8	74	94	99	111^1^	8	
Valine	Trt	166	174	199	241	15	181	207	231	258	15	205	311	356	351	15	204	254	282	306	15	0.53
	Ctl	164	175	201	238	15	198	217	207	209	17	201	292	341	347	15	186	230	266	311	16	

^1^Means within week by treatment are significantly different from fasted value (*P *≤ 0.05).

^2^Significantly different from the same timepoint in the same group at week −1 (*P* ≤ 0.05).

Differences were observed in serum Trp concentrations when compared to the same timepoint at week −1 within group. No differences were observed from week −1 at week 2 or 8 at any timepoint in either group (*P* > 0.05). At week 4, fasted levels of serum Trp in both Trt and Ctl were lower than the same timepoint at week −1 (*P* ≤ 0.05).

### Dispensable amino acids postprandial response

Mean serum dispensable AA (DAA) concentrations of Trt and Ctl at each week for all timepoints are reported in Table S1. There was no effect of treatment on any DAA concentrations, yet a treatment*week interaction effect was observed for Asn and GSH (Table S2). Serum Asn concentrations at week 8 were lower than week −1 in the Trt group (*P* ≤ 0.05) but not in the Ctl group (*P* > 0.05), and serum concentrations at week 2 were lower in the Trt group when compared to the Ctl group in the same week (*P* ≤ 0.05). Finally, serum GSH concentrations were lower at week 4 and week 8 when compared to week −1 in the Trt group (*P* ≤ 0.05), a difference not reflected in the Ctl group (*P* > 0.05). No treatment*week*timepoint interaction effect was observed for any DAA (*P* > 0.05).

### Interleukin-10

There was no treatment or treatment*week interaction effect for serum IL-10 concentrations (*P *> 0.05; data not shown).

### Case study

**Figure 1.**
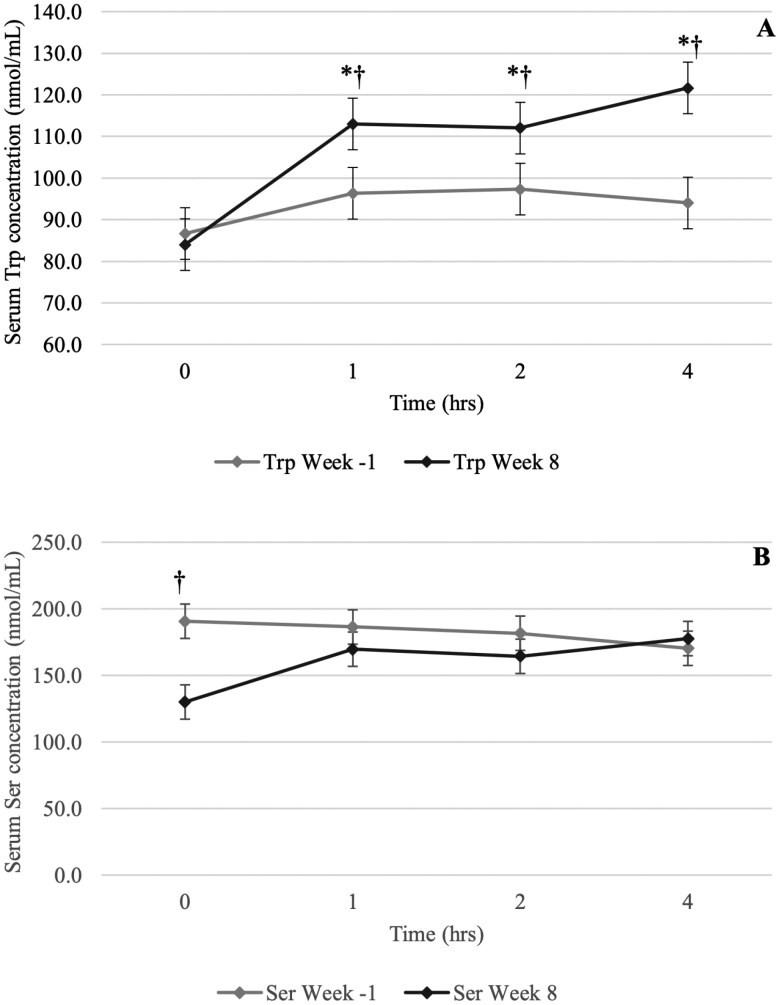
Mean serum concentrations (nmol/mL) of tryptophan (Trp; A) and serine (Ser; B) in 3 treatment dogs demonstrating elevated gut permeability at week −1 that was ameliorated by week 8. *means within week are significantly different from fasted (*P *≤ 0.05). ^†^significantly different from the same timepoint at week −1 (*P* ≤ 0.05).

For the 3 dogs identified from the Trt group as having greater intestinal permeability than the other dogs, there was an effect of week for all indispensable AA (IAA) except for Phe, as well as for Ala, Asn, Gln, Gly, Ser, and Tau, with a significant week*timepoint interaction effect observed for Trp and Ser. Data for the week effect on the IAA are shown in Table 2. When pooled across all timepoints within each week, serum concentrations of the IAA Ile, Leu, Lys, Met, Thr, Trp, and Val were greater at week 8 compared to week −1 (*P* ≤ 0.05), while serum His concentrations were greater at week −1 than at week 8 (*P* > 0.05). Data for the postprandial amino acid (AA) response at weeks −1 and 8 for IAA are shown in Fig. 1, and for dispensable AA (DAA) in Table S3. A significant week*timepoint interaction effect was observed for Trp (Fig. 1A) and Ser (Fig. 1B) (*P* ≤ 0.05), with no such interaction observed for any other IAA or DAA (*P* > 0.05). At week 8, serum concentrations of Trp were greater at 1, 2, and 4 h postprandial compared to the same timepoints at week −1 (*P* ≤ 0.05), while serum Ser concentrations were lower in the fasted state at week 8 compared to the same timepoint at week −1 (*P* ≤ 0.05).

**Table 2.** Mean pooled postprandial serum indispensable AA concentrations (nmol/mL) over a 10-week period in 3 treatment dogs demonstrating elevated gut permeability at week −1 that was ameliorated by week 8

Amino acid	Week −1	Week 8	SEM	*P*-value
Histidine	130^a^	120^b^	4	0.03
Isoleucine	82^b^	112^a^	2	≤0.01
Leucine	140^b^	200^a^	5	≤0.01
Lysine	177^b^	198^a^	27	0.02
Methionine	58^b^	70^a^	2	≤0.01
Phenylalanine	87	89	6	0.35
Threonine	164^b^	213^a^	10	≤0.01
Tryptophan	94^b^	108^a^	6	≤0.01
Valine	196^b^	274^a^	5	≤0.01

^a,b^Means within a row with different letters are significantly different (*P* ≤ 0.05).

## Discussion

To the authors’ knowledge, we are the first to demonstrate that in dogs with impaired gut permeability, brewers yeast supplementation can improve gut permeability (Rummell et al., 2022) and also increase the appearance of dietary AA in serum, suggesting increased absorption or decreased use of AA within the GIT. Though the initial hypothesis was disproven, supported by the lack of difference in serum IL-10 and the reduction in serum GSH over time, the results of this study as a whole suggest a benefit of yeast supplementation for dogs experiencing subclinical gastrointestinal (**GI**) dysfunction while having no detrimental effects in healthy dogs.

Indispensable AAs are either not synthesized or cannot be synthesized in sufficient quantities endogenously to support protein synthesis and other metabolic functions and therefore must be provided in the diet. To be utilized for metabolic functions, dietary AA must first be digested through enzymatic hydrolysis of proteins, followed by absorption via enterocytes into circulation (Stein et al., 2007). Therefore, impaired intestinal barrier function, which mediates nutrient absorption, can negatively affect AA uptake. Maintaining or restoring gut health enhances the surface area for nutrient absorption, which in turn increases circulating AA (Farré et al., 2020). Measuring postprandial AA concentrations provides valuable insights into gut barrier integrity and response to a meal. To our knowledge, no other studies have reported postprandial AA response in dogs supplemented with brewers yeast. However, numerous studies that measure digestibility, which underpins postprandial AA appearance, have been conducted in other species receiving yeast supplementation. When measured in yeast-supplemented pigs, postprandial AA concentrations are variable, though largely suggestive of a beneficial effect of the yeast supplement (Mateo and Stein, 2007; Moehn et al., 2010; Xiong et al., 2015; Wu et al., 2018; Park et al., 2021).

In this study, measurable differences were observed in postprandial AA concentrations in the test population, particularly for serum Trp, His, and GSH. Trp is involved in the gut–brain axis through 3 primary pathways: serotonin synthesis, the kynurenine pathway, and the microbial indole pathway (Roth et al., 2021). Trp is the only substrate for serotonin synthesis, which occurs predominantly in the distal GIT (Boadle-Biber, 1993), almost entirely by enterochromaffin cells (**ECs**) in the intestinal mucosa. Released serotonin impacts gut signaling (Gershon et al., 1977; Mawe and Hoffman, 2013), motility (Browning, 2015), intestinal homeostasis (Reigstad et al., 2015), vasoreactivity (Mohammad‐Zadeh et al., 2008), and immune cell activity (Herr et al., 2017). While most Trp oxidation via the kynurenine pathway occurs in the liver, a portion occurs in the brain and GIT (Roth et al., 2021). The activity of indoleamine 2,3-dioxygenase (**IDO**), the enzyme degrading Trp to kynurenine, is induced by proinflammatory cytokines and microbial components (Yi et al., 2015), and influenced by short chain fatty acids (He et al., 2013). Kynurenine crosses the blood—brain barrier affecting neural signaling. Lastly, gut bacteria expressing tryptophanase convert Trp to indole and its derivatives (e.g., indole-3-acetate, indole-3-propionic acid), which can attenuate inflammation (Ehrlich et al., 2020), activate aryl hydrocarbon receptors influencing immune responses (Esser et al., 2009), and inhibit dysbiosis (Zelante et al., 2013; Lamas et al., 2016).

Yeast supplementation, potentially through its documented effects on gut barrier function (Rummell et al., 2022), its influence on microbial populations, or its local immune-modulatory properties, could influence these Trp metabolic pathways. For instance, enhanced barrier integrity could increase Trp absorption across the enterocytes. Alternatively, alterations in the microbiome induced by yeast could shift the balance of Trp degradation versus microbial synthesis, or modulation of inflammatory cytokines (potentially reducing IDO induction) could decrease Trp catabolism via the kynurenine pathway. The importance of Trp availability for gut health, particularly during impaired intestinal integrity, is well-documented (Shizuma et al., 2009; Kim et al., 2010; Ooi et al., 2011; Hisamatsu et al., 2012; Liu et al., 2017; He et al., 2018), highlighting the potential significance of interventions that can enhance its postprandial appearance.

No studies have evaluated the effects of supplemental yeast on circulating Trp in dogs or other species. In dogs undergoing exercise training and supplemented with Trp to achieve a Trp to large neutral AA (LNAA; Tyr, Phe, Leu, Ile, Val) ratio of 0.075:1, higher concentrations of both fasted and postprandial Trp, Trp:LNAA, and serotonin were observed compared to a control group (Templeman et al., 2020). These dogs were also more likely to have improved stool quality (Templeman et al., 2020). While the dogs in the present study were not undergoing exercise training as part of the protocol, they did engage in regular exercise beginning in week 3 of the Trt period. Additionally, dogs in the current study showed reduced fasted serum Trp concentrations at week 4 compared to week −1. This decline coincides with the onset of their training season, suggesting increased utilization of Trp during this period. Interestingly, although the dogs in the case study group had lower fasted serum Trp concentrations at week 8 compared to baseline, all postprandial samples were higher than their fasted counterparts and when compared to the same timepoints at week −1. Therefore, the observed increase in postprandial serum Trp in the Trt group, especially in dogs starting with impaired permeability, likely reflects improved intestinal function potentially driven by yeast supplementation. This could stem primarily from enhanced absorption secondary to improved barrier integrity (Rummell et al., 2022), possibly coupled with reduced Trp utilization or altered microbial metabolism within the GIT influenced by the yeast. While no definitive conclusions can be drawn from the present study, further research into the effects of yeast supplementation in exercising dogs is needed to assess its impact on gut barrier function and nutrient absorption.

Serum His concentrations followed a similar pattern as Trp. Fasted concentrations were numerically lower at weeks 2, 4, and 8 compared to week −1, and postprandial concentrations at weeks 2, 4, and 8 were significantly greater than fasted concentrations in the Trt group. In the Ctl group, only weeks 4 and 8 increased significantly from week −1. Additionally, in the case study group, His was the only IAA where concentrations were reduced at week 8 compared to week −1.

Several mechanisms related to the brewers yeast supplement could contribute to these observed changes in postprandial His concentrations. Yeast supplementation modulates the gut microbial ecosystem (Beloshapka et al., 2013; Cui et al., 2024) which directly impacts luminal His metabolism. Gut bacteria utilize His via 2 primary routes: the catabolic Histidine Utilization (Hut) pathway, which degrades His into glutamate, ammonia, and formate/formamide for nutrient recovery, and the histidine decarboxylase (HDC) pathway, which converts His into the bioactive amine histamine (Bender, 2012; Schink et al., 2018). Yeast components, such as mannooligosaccharides and β-glucans, act as prebiotics and alter the abundance and activity of microbial groups that are responsible for degradation of His. For example, yeast products have been reported to decrease populations of *E. coli* (potentially reducing HDC activity from these species) while increasing *Lactobacillus* and *Bifidobacterium* (Beloshapka et al., 2013; Bastos et al., 2023). It is plausible that the specific microbial shifts induced by this brewers yeast supplement altered the balance of luminal His metabolism, potentially reducing the overall degradation via the Hut pathway or its conversion to histamine via the HDC pathway, thereby increasing the amount of intact His available for absorption.

Furthermore, the documented capability of yeast supplementation to improve gut barrier integrity in dogs (Rummell et al., 2022; Cui et al., 2024), possibly by strengthening tight junctions and reducing low-grade inflammation, could enhance the efficiency of His absorption. Restoring barrier integrity likely supports optimal function of the specialized, active transport systems responsible for transcellular His uptake across enterocytes (Kiela and Ghishan, 2016). Improved epithelial health fostered by yeast could facilitate more effective absorption.

While His itself plays immunoregulatory roles (Schink et al., 2018), and lower fasted levels have been reported in dogs with enteropathies (Benvenuti et al., 2020), the lack of significant difference in postprandial His concentrations across weeks in the Trt group suggest absorption may be influenced by baseline fasted levels, but overall greater postprandial rise compared to fasted levels points towards enhanced availability potentially mediated by yeasts combined effects on luminal metabolism and gut barrier function. To the authors knowledge, no studies have reported on the effects of yeast supplementation on circulating His in any species, highlighting the novelty of these findings and the need for further investigation into these yeast-mediated mechanisms.

Glutathione concentrations in weeks 4 and 8 were lower when pooled and compared to week −1 in the Trt group. This finding was somewhat unexpected, given that GSH is a key antioxidant (Shimada et al., 2015) crucial for maintaining cellular redox states (Jones, 2002), and *Saccharomyces cerevisiae* is recognized as a natural source of both GSH and its precursors, like S-acetyl-glutathione (SAG; Tahmasebi et al., 2016; Martello et al., 2023). Furthermore, in vitro experiments using porcine cell lines reported that yeast cell wall components could up-regulate GSH production under induced oxidative stress (Guo et al., 2019). However, the bioavailability of orally supplemented GSH is uncertain, and the in vitro results may not fully translate to the systemic in vivo response in healthy, non-stressed dogs.

Several mechanisms potentially driven by yeast supplementation could explain the observed decrease in circulating GSH. A key consideration is the interplay between GSH and the broader antioxidant enzyme network, which yeast is known to modulate. Supplementation with various yeast products has been reported to enhance the activity of endogenous antioxidant enzymes, including Glutathione Peroxidase (**GPx**) and Superoxide Dismutase (**SOD**) in canine and other models (Yu et al., 2021; Wilson et al., 2022; Cui et al., 2024). GPx utilizes GSH as a cofactor to neutralize hydrogen peroxide and lipid hydroperoxides, oxidizing GSH to GSSG in the process (Bhattacharyya et al., 2014; Martello et al., 2023). Therefore, a plausible yeast-mediated mechanism for lower circulating GSH is an upregulation of GPx activity. If the brewers yeast supplement stimulated GPx function, as reported in aged dogs (Cui et al., 2024), this would lead to increased consumption or turnover of GSH, potentially lowering the measured systemic concentrations, even in these clinically healthy dogs.

The influence of yeast on gut microbiota and associated gut health improvements (Rummell et al., 2022; Cui et al., 2024) adds complexity. Typically, improved gut health and reduced dysbiosis would be expected to decrease systemic oxidative stress (Sun et al., 2024; Steinert et al., 2025), which should spare GSH. The observed decrease suggests that either any systemic oxidative stress reduction was insufficient to counteract increased enzymatic utilization, or perhaps the process of microbial modulation and immune interaction initiated by the yeast supplement had transient metabolic costs involving GSH turnover. Additionally, the study dogs began regular exercise during the Trt period. Exercise is known to increase reactive oxygen species production and can lead to GSH depletion or increased turnover (Sechi et al., 2017). It is possible that the yeast supplementation interacted with the exercise stress, potentiating the adaptive antioxidant enzymatic response to exercise, leading to greater GSH utilization than would occur with exercise alone.

GSH deficiency is documented in disease and aging (Domínguez et al., 1998; Lyons et al., 2001; Rahman et al., 2004; Viviano et al., 2009). The dogs in the current study were considered healthy, and serum Cys, a rate-limiting precursor for GSH synthesis (Dickinson and Forman, 2002) did not significantly change, making drastically impaired synthesis an unlikely explanation based on available data. Therefore, the decreased GSH concentrations observed following brewers yeast supplementation are most likely attributable to increased utilization, potentially driven by yeast-induced upregulation of GSH-dependent enzyme activity interacting with the physiological demands of exercise, rather than a detrimental effect or impaired synthesis. Further investigations measuring specific antioxidant enzyme activities and the ratio of free and bound GSH alongside yeast supplementation are required to confirm these mechanisms.

In human studies of inflammatory conditions of the GIT, the integrity of the intestinal barrier can be impaired, which can result in pathogen penetration and malnutrition (Li et al., 2007; Landy et al., 2016; Bao et al., 2017; He et al., 2018; Vich Vila Arnau et al., 2018). Less research is available on these disease states in dogs, with only 1 study evaluating the effects of a yeast ß-glucan supplement on symptoms of inflammatory bowel disease (Rychlik et al., 2013). This study reported greater concentrations of IL-10 and lower concentrations of IL-6 in ß-glucan supplemented dogs after 42 d, supporting its efficacy in dogs experiencing inflammation in the GIT (Rychlik et al., 2013).

Alterations in both pro- and anti-inflammatory cytokine markers have been measured in dogs supplemented with yeast and its components (Rychlik et al., 2013), suggesting immune-modulatory effects of these carbohydrates. De La Guardia-Hidrogo et al. (2024) supplemented dogs with a functionalized canola meal containing varying yeast doses (low, medium, or high) and reported no differences between treatments in tumor necrosis factor-α but an increase in viable T-cells in the low-yeast group, indicating immune modulation. In the present study, no differences were measured in fasted serum IL-10, a major suppressor of the immune response and inflammatory activity by way of regulatory T-cell induction (Moore et al., 2001). Given the lack of substantial differences between the Trt and Ctl groups, we would not expect inflammatory cytokine markers to show significant variation.

Changes in gut morphology, potentially due to increased feed intake, exercise, or seasonal fluctuations, may contribute to the changes in AA concentrations observed. One study measured gut morphology of dogs and reported that dietary factors can influence intestinal morphology, with dogs consuming a plant-product based diet having a greater duodenal villus width and jejunal and ileal villus height compared to an animal-product based diet (Kuzmuk et al., 2005). Owing to the terminal nature of such work, many studies have investigated changes in microbial composition of the gut as a marker of gut function (Gagné et al., 2013; Tysnes et al., 2020; Belà et al., 2024; Wang et al., 2024). These studies generally report increased prevalence of enteropathogenic bacteria in exercising dogs not receiving gut health supporting supplements, notably *Enterobacteriaceae* which are known to be associated with dysbiosis and intestinal inflammation (Baldelli et al., 2021). While the investigated substance may impact results, diet clearly impacts the GIT ecosystem. In this study, all dogs consumed the same commercially available diet, and only fecal arabinose concentrations differed between the Trt and Ctl groups (Rummell et al., 2022). As previously reported, feed intake in the present study increased throughout the study period as a result of declining environmental temperatures and increased energy expenditure (Rummell et al., 2022). Increased feed intake could have affected gut morphology, enhancing nutrient absorption by increasing surface area. Additionally, the commercially available diet used in this study includes many ingredients that could positively influence gut health, such as chicory root, marshmallow root, turmeric, and many fibrous ingredients (Rummell et al., 2022). Overall, yeast is 1 ingredient of many commonly found in commercially available diets with known benefits to the GIT.

Exercise also impacts gut function by altering microbial populations and transit time, which in turn influences nutrient absorption. For example, in racing Alaskan Huskies, exercise increased intestinal mucosal barrier permeability (Royer et al., 2005). Similar results have been observed in other sled dog populations, with reduced fecal quality and increased dysbiosis-associated bacteria reported post-exercise (Gagné et al., 2013; Tysnes et al., 2020; Belà et al., 2024; Wang et al., 2024). While the dogs in this study participated in exercise, there were no differences in gut permeability pre- and post-treatment, suggesting that the exercise intensity may not have been sufficient to elicit a significant effect. There is a potential for an acute effect, that may have been noted at week 4 following the onset of exercise, however permeability was not assessed at that time so no definitive conclusions can be drawn.

Finally, environmental factors such as declining outdoor temperatures and increased energy expenditure may have contributed to some of the measured differences, as previously reported (Rummell et al., 2022). However, research on sled dogs housed outdoors year-round in Greenland reported only minor metabolic differences (Gerth et al., 2010), making this a less likely explanation for the changes observed in the present study.

While intriguing, it is important to note that the results of the case study must be viewed with caution. With only 3 dogs, and none from the control group, limited conclusions can be drawn regarding overall effects of yeast supplementation on gut permeability. Further research is needed to explore the efficacy of yeast in clinical conditions, perhaps within a population with known GI dysbiosis.

In conclusion, while few differences were observed between groups in this study, the data from the case study group support the inclusion of yeast supplements in commercially available diets for dogs targeted to support gut health. No detrimental effects were observed in healthy dogs, and dogs with elevated gut permeability showed improvement, with increased peripheral AA availability. Future studies should evaluate yeast supplementation in both healthy and diseased states and investigate yeast as a dietary component rather than a top-dress to determine potential effects related to processing.

## Supplementary Data

Supplementary data are available at *Journal of Animal Science* online.

skaf180_suppl_Supplementary_Materials

## Abbreviations:

AA: amino acid
AID: apparent ileal digestibility
AAFCO: Association of American Feed Control Officials
BW: bodyweight
Ctl: control
DAA: dispensable amino acid
ECs: enterochromaffin cells
ELISA: enzyme-linked immunosorbent assay
GI: gastrointestinal
GIT: gastrointestinal tract
GPx: glutathione peroxidase
HDC: histidine decarboxylase
IRE: immunosuppressant-responsive enteropathy
IAA: indispensable amino acid
IDO: indoleamine 2,3-dioxygenase
IL: interleukin
LNAA: large neutral amino acid
NRC: National Research Council
SAG: S-acetyl-glutathione
SOD: superoxide dismutase
Trt: treatment
UPLC: ultra-performance liquid chromatography

## Notes

*Conflict of interest statement.* L.M.R is an employee of Wilur-Ellis Nutrition. J.R.T. is an employee of Primal Pet Foods. C.L.C.F. is an employee of Champion Petfoods. A.K.S. is the Champion Petfoods Chair in Canine and Feline Nutrition, Physiology and Metabolism and additionally consults for Champion Petfoods. A.K.S. has received honoraria and research funding from various pet food manufacturers, ingredient suppliers, and national and provincial funding agencies.
