# Supplementary material for: The effects of supplemental brewers yeast on postprandial amino acid concentrations in healthy adult sled dogs
Source: J Anim Sci. 2025 May 24;103:skaf180. doi: 10.1093/jas/skaf180 (PMC12202307; doi:10.1093/jas/skaf180)
Supplement: skaf180_suppl_Supplementary_Materials [file skaf180_suppl_supplementary_materials.docx]

**Table S1.** Mean serum concentrations of dispensable AA (DAA; nmol/mL) in control and yeast-supplemented dogs over a 10-week period.

|  |  | Week -1 | | | | | Week 2 | | | | | Week 4 | | | | | Week 8 | | | | | *P*-value |
| --- | --- | --- | --- | --- | --- | --- | --- | --- | --- | --- | --- | --- | --- | --- | --- | --- | --- | --- | --- | --- | --- | --- |
| Amino Acid | Grp | fasted | 1 hr | 2 hr | 4 hr | SEM | fasted | 1 hr | 2 hr | 4 hr | SEM | fasted | 1 hr | 2 hr | 4 hr | SEM | fasted | 1 hr | 2 hr | 4 hr | SEM | Wk*Trt*Time |
| Alanine | Trt | 478 | 575 | 598 | 575 | 39 | 383 | 589 | 503 | 503 | 41 | 233 | 392 | 459 | 513 | 39 | 403 | 559 | 578 | 634 | 39 | 0.97 |
|  | Ctl | 537 | 572 | 582 | 552 | 41 | 482 | 565 | 541 | 519 | 47 | 266 | 367 | 491 | 517 | 41 | 381 | 555 | 529 | 590 | 44 |  |
| Arginine | Trt | 194 | 220 | 231 | 226 | 14 | 188 | 231 | 236 | 231 | 14 | 134 | 200 | 232 | 211 | 14 | 164 | 244 | 246 | 246 | 14 | 0.68 |
|  | Ctl | 219 | 245 | 261 | 240 | 14 | 218 | 241 | 236 | 238 | 16 | 144 | 199 | 231 | 253 | 15 | 175 | 246 | 274 | 286 | 15 |  |
| Asparagine | Trt | 50 | 68 | 78 | 88 | 9 | 52 | 44 | 47 | 40 | 9 | 35 | 72 | 93 | 79 | 9 | 33 | 58 | 51 | 66 | 9 | 0.84 |
|  | Ctl | 47 | 67 | 70 | 83 | 9 | 62 | 69 | 58 | 74 | 11 | 35 | 63 | 84 | 104 | 9 | 41 | 49 | 54 | 68 | 10 |  |
| Aspartate | Trt | 12 | 11 | 13 | 15 | 1 | 10 | 12 | 13 | 16 | 1 | 7 | 16 | 16 | 19 | 1 | 8 | 11 | 14 | 16 | 1 | 0.35 |
|  | Ctl | 10 | 11 | 13 | 14 | 1 | 11 | 13 | 12 | 12 | 2 | 7 | 11 | 15 | 18 | 1 | 9 | 12 | 14 | 18 | 2 |  |
| Cysteine | Trt | 521 | 687 | 680 | 506 | 97 | 452 | 453 | 439 | 437 | 76 | 365 | 358 | 343 | 355 | 60 | 486 | 477 | 449 | 465 | 69 | 0.97 |
|  | Ctl | 444 | 503 | 479 | 502 | 76 | 506 | 515 | 490 | 460 | 85 | 396 | 377 | 362 | 362 | 65 | 553 | 494 | 491 | 508 | 70 |  |
| Glutamine | Trt | 907 | 847 | 777 | 739 | 44 | 798 | 779 | 759 | 742 | 46 | 684 | 691 | 699 | 687 | 44 | 797 | 756 | 692 | 723 | 44 | 0.5 |
|  | Ctl | 856 | 820 | 770 | 690 | 46 | 770 | 756 | 793 | 698 | 51 | 647 | 558 | 602 | 750 | 44 | 822 | 725 | 718 | 781 | 48 |  |
| Glutamate | Trt | 94 | 88 | 90 | 92 | 3 | 89 | 85 | 92 | 95 | 3 | 64 | 72 | 82 | 90 | 3 | 83 | 85 | 86 | 92 | 3 | 0.53 |
|  | Ctl | 95 | 89 | 91 | 91 | 3 | 89 | 89 | 88 | 84 | 4 | 64 | 72 | 83 | 93 | 3 | 81 | 84 | 85 | 94 | 4 |  |
| Glutathione^1^ | Trt | 9 | 14 | 14 | 13 | 2 | 9 | 10 | 10 | 11 | 1 | 9 | 10 | 10 | 11 | 1 | 10 | 11 | 11 | 10 | 1 | 0.95 |
|  | Ctl | 8 | 11 | 12 | 11 | 2 | 10 | 11 | 10 | 11 | 2 | 11 | 11 | 11 | 12 | 1 | 12 | 14 | 13 | 14 | 1 |  |
| Glycine | Trt | 270 | 320 | 352 | 343 | 18 | 241 | 314 | 314 | 323 | 19 | 152 | 261 | 311 | 345 | 18 | 201 | 301 | 308 | 345 | 18 | 0.51 |
|  | Ctl | 267 | 324 | 347 | 346 | 19 | 267 | 329 | 296 | 308 | 22 | 150 | 251 | 324 | 391 | 19 | 205 | 332 | 359 | 402 | 20 |  |
| Homocysteine^1^ | Trt | 19 | 30 | 26 | 17 | 5 | 18 | 17 | 16 | 15 | 4 | 15 | 14 | 13 | 12 | 3 | 19 | 19 | 17 | 16 | 3 | 0.95 |
|  | Ctl | 18 | 18 | 17 | 15 | 5 | 17 | 17 | 14 | 13 | 4 | 16 | 14 | 13 | 12 | 3 | 21 | 19 | 17 | 16 | 3 |  |
| Proline | Trt | 122 | 188 | 224 | 249 | 13 | 136 | 218 | 223 | 245 | 14 | 82 | 184 | 241 | 268 | 13 | 106 | 219 | 241 | 268 | 13 | 0.52 |
|  | Ctl | 128 | 191 | 227 | 251 | 14 | 165 | 223 | 195 | 219 | 14 | 81 | 181 | 247 | 288 | 14 | 109 | 221 | 259 | 303 | 15 |  |
| Serine | Trt | 174 | 174 | 173 | 172 | 13 | 120 | 168 | 152 | 152 | 14 | 105 | 167 | 173 | 174 | 13 | 127 | 167 | 166 | 180 | 13 | 0.88 |
|  | Ctl | 151 | 158 | 164 | 157 | 14 | 126 | 137 | 154 | 154 | 16 | 102 | 130 | 157 | 192 | 14 | 134 | 170 | 182 | 204 | 15 |  |
| Taurine | Trt | 112 | 121 | 152 | 125 | 12 | 135 | 144 | 146 | 134 | 13 | 135 | 191 | 178 | 134 | 12 | 159 | 184 | 180 | 160 | 12 | 0.32 |
|  | Ctl | 122 | 129 | 152 | 145 | 13 | 155 | 156 | 155 | 132 | 14 | 135 | 158 | 180 | 169 | 13 | 170 | 215 | 195 | 181 | 13 |  |
| Tyrosine | Trt | 67 | 72 | 74 | 73 | 4 | 56 | 71 | 74 | 71 | 4 | 59 | 77 | 82 | 75 | 4 | 55 | 81 | 82 | 76 | 4 | 0.96 |
|  | Ctl | 67 | 69 | 70 | 73 | 4 | 64 | 69 | 69 | 67 | 4 | 63 | 77 | 80 | 80 | 4 | 60 | 78 | 82 | 83 | 4 |  |

^1^Values were computed in a separate analysis from the other amino acids (AAs).

**Table S2.** Mean serum concentrations of dispensable AA (DAA; nmol/mL) pooled within week in control and yeast-supplemented dogs.

| Amino Acid | Grp | Week -1 | Week 2 | Week 4 | Week 8 | SEM | *P*-value |
| --- | --- | --- | --- | --- | --- | --- | --- |
| Alanine | Trt | 556 | 496 | 399 | 543 | 25 | 0.46 |
|  | Ctl | 561 | 527 | 410 | 514 | 27 |  |
| Arginine | Trt | 218 | 222 | 194 | 225 | 9 | 0.74 |
|  | Ctl | 241 | 233 | 207 | 245 | 10 |  |
| Asparagine | Trt | 71 | 46^†^ | 70 | 52* | 5 | ≤ 0.01 |
|  | Ctl | 67 | 66 | 72 | 53 | 5 |  |
| Aspartate | Trt | 12 | 13 | 14 | 12 | 1 | 0.17 |
|  | Ctl | 12 | 12 | 13 | 13 | 1 |  |
| Cysteine | Trt | 598 | 445 | 355 | 469 | 38 | 0.07 |
|  | Ctl | 482 | 493 | 374 | 511 | 42 |  |
| Glutamine | Trt | 817 | 769 | 690 | 742 | 31 | 0.3 |
|  | Ctl | 784 | 754 | 639 | 762 | 33 |  |
| Gluathione^1^ | Trt | 12 | 10 | 10* | 11* | 1 | ≤ 0.01 |
|  | Ctl | 10 | 10 | 11 | 13 | 1 |  |
| Glutamate | Trt | 91 | 90 | 77 | 87 | 2 | 0.61 |
|  | Ctl | 91 | 88 | 78 | 86 | 3 |  |
| Glycine | Trt | 321 | 298 | 267 | 289 | 13 | 0.13 |
|  | Ctl | 321 | 300 | 279 | 324 | 13 |  |
| Homocysteine^1^ | Trt | 23 | 16 | 13 | 18 | 2 | 0.15 |
|  | Ctl | 17 | 15 | 14 | 18 | 2 |  |
| Proline | Trt | 196 | 205 | 194 | 208 | 8 | 0.58 |
|  | Ctl | 199 | 201 | 199 | 223 | 8 |  |
| Serine | Trt | 173 | 148 | 155 | 160 | 9 | 0.15 |
|  | Ctl | 158 | 143 | 145 | 172 | 9 |  |
| Taurine | Trt | 127 | 140 | 159 | 171 | 8 | 0.43 |
|  | Ctl | 137 | 150 | 160 | 190 | 9 |  |
| Tyrosine | Trt | 71 | 68 | 73 | 73 | 3 | 0.66 |
|  | Ctl | 70 | 68 | 75 | 76 | 3 |  |

^1^Values were computed in a separate analysis from the other amino acids (AAs).

*****Means within group are significantly different from week -1 (*P* ≤ 0.05).

^†^Means within week are significantly different from Control (*P* ≤ 0.05).

**Table S3.** Mean serum concentrations of dispensable AA (DAA; nmol/mL) in 3 treatment dogs demonstrating elevated gut permeability at week -1 that was ameliorated by week 8.

|  | Week -1 | | | | | Week 8 | | | | | *P*-value |
| --- | --- | --- | --- | --- | --- | --- | --- | --- | --- | --- | --- |
| Amino Acid | fasted | 1 hr | 2 hr | 4 hr | SEM | fasted | 1 hr | 2 hr | 4 hr | SEM | Wk*Time |
| Alanine | 550 | 678 | 651 | 657 | 65 | 337 | 616 | 582 | 704 | 65 | 0.12 |
| Arginine | 179 | 225 | 227 | 221 | 8 | 163 | 242 | 242 | 241 | 8 | 0.08 |
| Asparagine | 52 | 76 | 81 | 86 | 17 | 34 | 56 | 59 | 67 | 17 | 1.00 |
| Aspartate | 17 | 12 | 14 | 16 | 3 | 9 | 12 | 14 | 17 | 3 | 0.33 |
| Cysteine | N/A^2^ | 463 | 477 | 462 | 69 | 472 | 496 | 450 | 487 | 86 | 0.89 |
| Glutamine | 907 | 834 | 787 | 733 | 40 | 797 | 720 | 642 | 680 | 40 | 0.63 |
| Glutamate | 89 | 85 | 86 | 92 | 6 | 87 | 89 | 87 | 93 | 6 | 0.97 |
| Homocysteine^1^ | N/A^2^ | 21 | 20 | 18 | 1 | 18 | 19 | 16 | 15 | 1 | 0.80 |
| Glycine | 254 | 327 | 340 | 328 | 20 | 194 | 298 | 294 | 323 | 28 | 0.39 |
| Glutathione^1^ | N/A^2^ | 11 | 12 | 13 | 2 | 10 | 11 | 11 | 10 | 2 | 0.27 |
| Proline | 121 | 207 | 227 | 250 | 14 | 105 | 220 | 230 | 254 | 14 | 0.58 |
| Serine | 191 | 186 | 182 | 170 | 13 | 130.0^†^ | 170 | 164 | 178 | 13 | 0.05 |
| Taurine | 114 | 122 | 169 | 127 | 23 | 174 | 175 | 179 | 156 | 23 | 0.69 |
| Tyrosine | 62 | 70 | 70 | 69 | 4 | 53 | 80 | 79 | 75 | 4 | 0.06 |

^1^Values were computed in a separate analysis from the other amino acids (AAs).

^2^Datapoints not available due to insufficient sample volume for analysis.

*means within week are significantly different from fasted (*P* ≤ 0.05).

^†^significantly different from the same timepoint at week -1 (*P* ≤ 0.05).
